# Prospects of evolution-based artificial intelligence models in genome-wide studies to stratify genetic risk variants in nonalcoholic fatty liver disease

**DOI:** 10.1097/MS9.0000000000000743

**Published:** 2023-05-08

**Authors:** Helen Huang, Wireko A. Awuah, Tulika Garg, Jyi Cheng Ng, Aashna Mehta, Krishna Ramamoorthy, Jacob Kalmanovich, Mohammad M. Hasan

**Affiliations:** a Royal College of Surgeons in Ireland, Faculty of Medicine and Health Science, Dublin, Ireland; b Sumy State University, Sumy, Ukraine; c Government Medical College and Hospital, Chandigarh, India; d Faculty of Medicine and Health Sciences, University of Putra Malaysia, Serdang, Malaysia; e Faculty of Medicine, University of Debrecen, Debrecen, Hungary; f Department of Biochemistry and Microbiology, Rutgers University-New Brunswick, New Jersey; g Drexel University College of Medicine, Philadelphia, Pennsylvania, USA; h Department of Biochemistry and Molecular Biology, Faculty of Life Science, Mawlana Bhashani Science and Technology University, Tangail, Bangladesh

**Keywords:** artificial intelligence, genetics, machine-learning, natural language processing, nonalcoholic fatty liver disease

## Abstract

The emergence of genome-wide association studies (GWAS) has identified genetic traits and polymorphisms that are associated with the progression of nonalcoholic fatty liver disease (NAFLD). Phospholipase domain-containing 3 and transmembrane 6 superfamily member 2 are genes commonly associated with NAFLD phenotypes. However, there are fewer studies and replicability in lesser-known genes such as LYPLAL1 and glucokinase regulator (GCKR). With the advent of artificial intelligence (AI) in clinical genetics, studies have utilized AI algorithms to identify phenotypes through electronic health records and utilize convolution neural networks to improve the accuracy of variant identification, predict the deleterious effects of variants, and conduct phenotype-to-genotype mapping. Natural language processing (NLP) and machine-learning (ML) algorithms are popular tools in GWAS studies and connect electronic health record phenotypes to genetic diagnoses using a combination of international classification disease (ICD)-based approaches. However, there are still limitations to machine-learning - and NLP-based models, such as the lack of replicability in larger cohorts and underpowered sample sizes, which prevent the accurate prediction of genetic variants that may increase the risk of NAFLD and its progression to advanced-stage liver fibrosis. This may be largely due to the lack of understanding of the clinical consequence in the majority of pathogenic variants. Though the concept of evolution-based AI models and evolutionary algorithms is relatively new, combining current international classification disease -based NLP models with phylogenetic and evolutionary data can improve prediction accuracy and create valuable connections between variants and their pathogenicity in NAFLD. Such developments can improve risk stratification within clinical genetics and research while overcoming limitations in GWAS studies that prevent community-wide interpretations.

## Introduction

Highlights

- Novel genome-wide association studies have extensively studied the genetic risk variants in nonalcoholic fattyl disease (NAFLD).
- Applications of artificial intelligence can improve NAFLD phenotyping from electronic health record data.
- The clinical consequences of most pathogenic genetic variants are still undetermined.
- Evolution-based artificial intelligence algorithms can improve genetic risk stratifications in NAFLD.

Nonalcoholic fatty liver disease (NAFLD), one of the most common diseases in the world, is characterized by adipose tissue deposition in the liver^1^. NAFLD is often associated with a sedentary lifestyle, a high-fat diet, and obesity; as a result, the most common ‘cure’ for NAFLD is weight loss. Scientific evidence suggests that NAFLD can contribute to insulin resistance and dyslipidemia and has been correlated with the development of diabetes and the metabolic syndrome^1^. Most people with NAFLD suffer from the less complicated form, steatosis, which is simply defined by the accumulation of fat in the liver. A certain percentage of patients may suffer from Nonalcoholic steatohepatitis (NASH), the more progressive form of NAFLD, which can result in liver cirrhosis, and the accumulation of scar tissue can eventually render the liver damaged beyond repair. If NAFLD and NASH were left untreated, extensive cirrhotic and fibrotic changes can eventually progress to hepatocellular carcinoma (HCC), treatment for which remains difficult. Amongst patients with both NASH and cirrhosis, the risk of developing HCC is high (12.8%) over a time period of 3 years^2^.

While the development of HCC is multifactorial, increased iron absorption in NASH, insulin resistance, and certain genetic polymorphisms are observed to have an important role in its pathophysiology^3,4^. Improvements in the prognosis of end-stage liver cancer requires robust identification strategies across NAFLD and NASH patients earlier in their care. As a result, the heritability of NAFLD has emerged to become a popular area of clinical research and has been extensively studied in genome-wide association studies (GWAS). Genome studies have linked various Single-Nucleotide Polymorphisms on genes such as Phospholipase domain-containing 3 (PNPLA3) and MBOAT7 that are associated with hepatic steatosis, fibrotic changes of the liver, and the acceleration of HCC development in patients with metabolic syndrome (i.e. insulin resistance, hypertension, obesity, etc.)^5^. Evolution-based artificial intelligence (AI) has emerged as a new technological model, which aims to improve variant identification of NAFLD-associated genes. However, the lack of risk stratification algorithms that could apply to international communities makes it challenging to accurately predict NAFLD-associated variants across ancestries. This review highlights the promising role of evolution-based AI in genome-wide studies to stratify genetic risk variants in NAFLD progression, improve risk stratification, and influence therapeutic implications.

## The emergence of GWAS in NAFLD

Several environmental and genetic factors play a significant role in the heritability of NAFLD, ranging from 22 to 50%^6^.

GWAS is a research strategy to discover genetic variations statistically related to disease risk. In this approach, the genomes of numerous individuals are scanned to identify genetic variants that are more prevalent in people with the disease or traits than those without the disease. These genomic variants are often used to locate neighboring variants that are directly responsible for the disease or trait once they have been found. Across GWAS studies investigating NAFLD severity, the condition is often identified based on visceral fat content and histological fibrosis severity to accurately represent steatosis in populations across clinical databases^7,8^. With consistent improvements in the definition of NAFLD and NASH, GWASs have greatly improved our understanding on its etiologies and pathophysiology in at risk patients with metabolic phenotypes^9^. There have been several studies linking nonsynonymous single-nucleotide polymorphisms in PNPLA3 and, more recently, Transmembrane 6 Superfamily member 2 (rs58542926), which was originally associated with the nearby neurocan (NCAN) gene^7,8,10^. Following subsequent investigations, it has been determined that both genetic relationships are associated with clinically significant outcomes, including steatohepatitis grade, cirrhosis or hepatic fibrosis stage, and, in the case of PNPLA3, hepatocellular carcinoma in NAFLD patients^11–13^.

While variations in or near LYPLAL1 and PNPLA3 do not affect serum lipid levels, those in or near neurocan (NCAN), glucokinase regulator (GCKR), and PPP1R3B do. Glycemic characteristics are also impacted by variations close to GCKR and PPP1R3B. Thus, it demonstrates the genetic basis of NAFLD and increases the variety of common genetic variations associated with this characteristic^7–10^. Recent studies using exome sequencing have correlated elevated alanine amino transferase (ALT) with an HSD17B13 SNP (rs6834314) in a general patient population and further demonstrated that this polymorphism is linked to NAFLD. This is widely supported by two more investigations^14,15^. In addition to the PNPLA3 effect, the GWAS suggests that IL17RA and other biologically significant genes play a significant role in the severity of NAFLD^16^.

## Commonly reported gene variants that shape liver phenotypes in NAFLD

A case-only GWAS using standardized liver enzymes as a quantitative phenotype found a strong association between the PNPLA3 risk allele and higher ALT levels. The highest effect was found for ALT at rs738409, with a *P*-value of 4.68 10 7^17^. Both AST and ALT were found to share a new impact at 2p22 close to the Xanthine Dehydrogenase (XDH) gene, which are expressed in the liver and involved in the oxidative metabolism of purines. This enzyme catalyzes the conversion of xanthine to uric acid and hypoxanthine to xanthine. Therefore, XDH-produced uric acid and reactive oxygen species may result in inflammation and oxidative stress. Recent research has demonstrated a correlation between the serum level of XDH and blood metabolic markers associated with obesity, including triglycerides, cholesterol, and glucose^18^. The transmembrane heparan sulphate proteoglycan Syndecan-1 (CD138, SDC1), which is abundantly expressed in the liver, also exerts metabolic effects and is suggested to cause a strong impact on liver enzyme levels; previous studies have corroborated with this hypothesis given that NAFLD patients exhibit higher serum levels of syndecan-1^19^. In addition to these genes, recent reports have also suggested that HSD17B13 is relevant to NAFLD through several genetic polymorphisms linked to lower risks of steatosis^15,20^.

Contrary to PNPLA3 and LYPLAL1, variants near NCAN (which encodes for an adhesion molecule), PPP1R3B (which encodes for a protein that regulates glycogen breakdown), and GCKR (which, by inhibiting glucokinase, regulates glucose storage/disposal and provides substrates for de novo lipogenesis) are linked to distinct alterations in serum and liver lipids as glycemic traits^21,22^. Certain polymorphisms in PNPLA3 and LYPLAL1 are known to induce hepatic steatosis without the presence of common metabolic characteristics such as insulin resistance and obesity in patient groups. These distinct correlation patterns suggest that hepatic steatosis is potentially regulated by various metabolic pathways, and is not just limited to lipid dysregulation or oxidative stress to the liver. Addressing genetic variation in the etiology of hepatic steatosis may create new avenues for individualized treatments, but there is a growing need to improve the accuracy and efficiency of these identification studies with modern technology.

## The influence of AI in clinical genetics research

AI has wide-ranging applications in clinical practice and stimulates human intelligence in computer-based networks. In the broad scheme of medicine, AI has been commonly known to maintain electronic health records (EHR) and assist patient consults, while machine-learning has been implemented to facilitate surgical procedures and assist in radiographic diagnostics^23^. In clinical genomics, AI computer systems can replicate applications requiring human intelligence through deep learning algorithms, thus improving image-based diagnostics, EHR phenotyping, and genetic variant classifications. Examples of these algorithms include computer vision (image acquisition), time-series analyses, automatic speech recognition, and Natural Language Processing (NLP)^24^.

**Figure 1:**
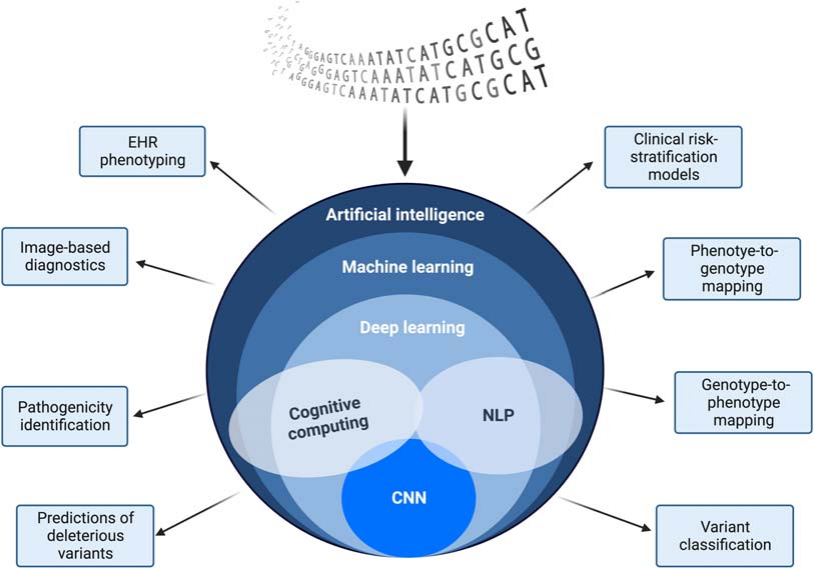
Hierarchical schematic of the artificial intelligence system and networks with specific applications to clinical genetics research. (Original work created with biorender.com by HH). EHR, Electronic Health Record; NLP, Natural Language Processing; CNN, Computer Neural Networks.

Convolution neural networks (CNN) drive the use of these AI algorithms, which are computational systems that consist of artificial neurons that take input data based on their trained interpretations. CNN allows for deep learning by interpreting features (i.e. diagnostics, imaging, procedure codes, and sequencing data) from complex datasets to drive genomic research. Though reports have found certain AI algorithms are limited and impractical compared with human intelligence, there have been major developments and improvements in CNN-based algorithms for genomics analyses. A review led by Dias in 2019 best summarizes adaptations that address the limitations of current AI-algorithm including deep variant caller algorithms to improve the accuracy of variant identification in a genome, combined annotation dependent depletion approach algorithms to predict deletions of variants. The use of primate AI to identify known pathogenic variants using cross-species analyses, and other CNN-based applications that would improve phenotype-to-genotype mapping and vice-versa ^24^. A summary of AI applications to genetics research is shown in Figure 1.

## Strengths and Limitations to NLP and ML Algorithms in NAFLD variant identification

NLP is one of the most commonly utilized AI-based algorithms that can extract important clinical information from EHRs, such as biopsies or images, and provide a molecular diagnosis with a combination of genotypic and phenotypic information. In particular, AI-based NLP approaches are often used to connect EHR phenotypes to genetic diagnoses through hierarchical statistical models and are often used for phenotype-to-genotype diagnostics. By detecting certain terms from the natural human language, NLP-based algorithms can computationally extract documents from phenotypic inputs and derive genetic variants of conditions. In NAFLD, the use of NLP in GWAS studies has risen in popularity and use, as NLP has shown an accuracy in detecting image-proven and biopsy-proven steatosis. A recent study led by Van Vleck supported these findings. NLP performed better in identifying NAFLD based on radiology notes and fibrosis scores from biopsies compared to traditional approaches using ICD-based approaches^25^. Given the propensity of NLP use in studies, multiple ML algorithms have been employed to identify the severity of NAFLD and its risk of progression to advanced disease such as cirrhosis. The Path AI research platform established in Boston trained a deep neural network to interpret over 600 liver biopsies and showed high concordance with liver histopathological interpretations made by trained pathologists. Such algorithms have also shown wide applications in identifying steatohepatitis from imaging, analyzing liver biomarkers such as ALT or gamma glutamyl transferase, and fibrosis staging^26,27^. As a result, AI has bridged several gaps in the knowledge of NAFLD progression, especially in risk stratification.

Despite major steps in identifying NAFLD variants underlying disease pathogenicity and progression, limitations of ML and NLP-based algorithms have been overlooked and may limit the strength of studies and there is still more room for growth in efficient algorithms that would stratify the risk of developing advanced liver disease based on genetic variants in studies. Current developments in AI models may be efficient, but the clinical consequences of over 98% of pathogenic variants in disease-associated genes have not yet been defined^28–30^. This may be the root cause of common limitations in NAFLD GWAS studies, such as underpowered sample sizes and the lack of replication due to the unknown association of certain variants^31^. As a result, most GWAS studies are limited to small cohorts that may not accurately represent the risks detected in populations. Data from EHRs have the potential for errors in lab measures and clinical diagnoses despite studies adjusting for confounding factors. This may also affect the clinical accuracy of quantitative liver enzyme tests that are not entirely specific to NAFLD, thus increasing the need for larger sample sizes and replication^31^.

Given that the NAFLD population is at a higher risk of developing HCC, it is crucial to identify variants that may increase the risk of irreversible advanced-stage liver cirrhosis^32^. Certain proposed models include genotype-to-phenotype predictions, which can improve risk stratification of NAFLD progression to HCC. However, current models are not equipped to predict the risk of developing HCC based on genotypes associated with liver phenotypes and do not represent causal relationships between risk factors. The accurate prediction of phenotypes from genotypes poses a huge problem, as the effects of genetic mutations are highly variable and can pose a barrier in personalized risk stratification for NAFLD.

## Evolution-based AI models in detecting genetic variants and applicability to NAFLD GWAS

**Figure 2:**
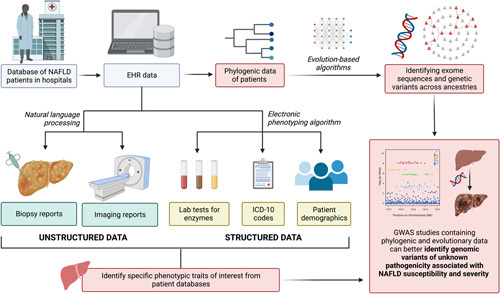
A general schematic demonstrating the process of extracting phenotypic data with current AI-based state-of-the-art algorithms (i.e. NLP) and the potential applications of evolution-based artificial intelligence models to analyze phylogenic data in GWAS studies. (Original work created with biorender.com by HH). EHR, Electronic Health Record; ICD-10, International Classification of Diseases 10th Revision; GWAS, Genome-Wide Association Studies; NAFLD, Nonalcoholic Fatty Liver Disease.

Evolution-based AI models are a topic of interest, as traditional state-of-the-art models rely on known disease labels such as ICD codes, which are subject to inaccuracies. By combining genomic information with evolutionary and phylogenetic data (seen in Fig. 2), trained algorithms can determine the propensity of disease-causing genes in their progression to disease-associated carcinomas and improve risk stratification within clinical genetics. One example of an evolution-based AI model is the evolutionary model of variant effect (EVE), a project developed in collaboration with the University of Oxford, Harvard, and the Broad Institute to determine genetic variants of significance related to a genetic condition^33^. By predicting the pathogenicity of over 36 million variants in over 3000 disease-associated genes, it was suggested that evolutionary information across species and ancestries provide better evidence on single variants tied with clinical phenotypes compared to traditional models. Experimental results of EVE disseminated the function of variants in BRCA1, TP53, MSH2, and PTEN with known disease links, accurately predicting the function of variants^34^.

Though EVE is currently the only evolution-based AI model created, evolutionary algorithms can supplement the function of pre-existing ML models and optimize its function in interpreting the risk of disease. In a study conducted by Ordikani, an evolutionary ML algorithm utilizes a ‘genetic algorithm’, an optimizer that is trained to evolve each proposed solution to reach the optimal outcome that can determine the risk of cardiovascular disease amongst patients in the Isfahan Cohort^35^. The effort in estimating the occurrence of cardiovascular disease used a risk assessment model called the eXplainable Persian Atherosclerotic cardiovascular disease Risk Stratification, and the algorithm was proven to have higher prediction accuracy than traditional chart models, even in the absence of quantitative tests. The study opened up the possibility of utilizing Darwin’s theory of evolution to conduct risk prediction tests that may overpower the functionalities of statistical models currently being used in genetic algorithms. Multilevel selection genetic algorithms is also an example of using evolutionary theory to use different reproduction models that compare the behavior of multiple variants through fitness functions. A comparison of variants can adjust to the diversity within a population between generations and can be utilized for different conditions^36^.

We propose that evolution-based AI models can improve the current limitations in NAFLD GWAS studies. Given the variability in hepatic phenotypes within communities and risk factors that increase the risk of progression of NAFLD, AI models, and evolutionary algorithms can provide better associations of risk factors in genotype-to-phenotype studies and improve risk-stratification models in liver diseases. These models can shed insight on whether certain genetic variants in identified NAFLD patients increase the risk of advanced-stage liver disease and subsequent cancer, given that there is a lack of literature surrounding the genetic risk of HCC development. In future studies, newer models can provide therapeutic avenues in personalized treatment within communities and better insight into rare variants with unknown pathogenicity that traditional methods do not address.

## Conclusion

As NAFLD patients remain at higher risk of developing HCC, it is imperative to uncover the pathogenicity of rare variants and improve early disease management. However, the current AI models are not capable of predicting the risk of developing HCC based on genotypes associated with liver phenotypes, hindering personalized risk stratification for NAFLD. AI and ML, with the combination of evolutionary data, hold great promise in optimizing GWAS studies, improving current limitations, and augmenting subsequent identification of genetic variants correlated with an increased risk of developing the NAFLD phenotype. Therefore, we propose that future studies consider evolution-based AI models, which can create valuable connections between genetic variants and the risk of diseases progressing to advanced-stage conditions. Further translational research exploring the utility of evolution-based AI models in GWAS studies can provide valuable evidence about its applications to NAFLD and its influence on patient outcomes.

## Ethical approval

NA.

## Consent

NA.

## Sources of funding

Authors have no funding to declare.

## Author contribution statement

All authors under the guidance of H.H.: substantial contribution to the conception and design of the work; all authors under the guidance of H.H. and W.A.A.: drafting the work and critical revision. All the authors read and approved the final version of the manuscript.

## Conflicts of interest disclosure

Authors have no conflicts of interest to declare.

## Research registration unique identification number (UIN)

1. Name of the registry: NA.
2. Unique Identifying number or registration ID: NA.
3. Hyperlink to your specific registration (must be publicly accessible and will be checked): NA.

## Guarantor

Mohammad Mehedi Hasan, Department of Biochemistry and Molecular Biology, Faculty of Life Science, Mawlana Bhashani Science and Technology University, Tangail, Bangladesh. e-mail: mehedi.bmb.mbstu@gmail.com (MMH)

## Data availability statement

No data available.

## Provence and peer review

Not commissioned, externally peer-reviewed.
